# Dietary Fiber-Derived Microbial Butyrate Suppresses ILC2-Dependent Airway Inflammation in COPD

**DOI:** 10.1155/2024/6263447

**Published:** 2024-07-09

**Authors:** Min Jiang, Jing Wang, Zheng Li, Dan Xu, Jing Jing, Fengsen Li, Jianbing Ding, Qifeng Li

**Affiliations:** ^1^ Xinjiang Key Laboratory of Respiratory Disease Research Traditional Chinese Medical Hospital of Xinjiang Uygur Autonomous Region, Urumqi 830011, Xinjiang, China; ^2^ Department of Immunology College of Basic Medicine Xinjiang Medical University, Urumqi 830011, Xinjiang, China; ^3^ Xinjiang Institute of Pediatrics Xinjiang Hospital of Beijing Children's Hospital Children's Hospital of Xinjiang Uygur Autonomous Region, Urumqi 830011, Xinjiang, China

## Abstract

Group 2 innate lymphoid cells (ILC2) strongly modulate COPD pathogenesis. However, the significance of microbiota in ILC2s remains unelucidated. Herein, we investigated the immunomodulatory role of short-chain fatty acids (SCFAs) in regulating ILC2-associated airway inflammation and explores its associated mechanism in COPD. In particular, we assessed the SCFA-mediated regulation of survival, proliferation, and cytokine production in lung sorted ILC2s. To elucidate butyrate action in ILC2-driven inflammatory response in COPD models, we administered butyrate to BALB/c mice via drinking water. We revealed that SCFAs, especially butyrate, derived from dietary fiber fermentation by gut microbiota inhibited pulmonary ILC2 functions and suppressed both IL-13 and IL-5 synthesis by murine ILC2s. Using *in vivo* and *in vitro* experimentation, we validated that butyrate significantly ameliorated ILC2-induced inflammation. We further demonstrated that butyrate suppressed ILC2 proliferation and GATA3 expression. Additionally, butyrate potentially utilized histone deacetylase (HDAC) inhibition to enhance NFIL3 promoter acetylation, thereby augmenting its expression, which eventually inhibited cytokine production in ILC2s. Taken together, the aforementioned evidences demonstrated a previously unrecognized role of microbial-derived SCFAs on pulmonary ILC2s in COPD. Moreover, our evidences suggest that metabolomics and gut microbiota modulation may prevent lung inflammation of COPD.

## 1. Introduction

Chronic obstructive pulmonary disease (COPD) is the third ranking contributor to global mortality, and it is characterized by persistent, progressive, and aggravated airflow obstruction, chronic lung inflammation, lung morphological alterations, and lung parenchymal destruction [1, 2]. Chronic persistent airway inflammation is a potential modulator of COPD pathology. Moreover, immune cells also contribute to COPD occurrence [3]. Likewise, group 2 innate lymphoid cells (ILC2s) release type 2 cytokines, which promote a wide range of inflammatory airway diseases, such as, allergic, asthma, and COPD diseases [4, 5].

ILC2s originate from lymphoid progenitor cells, and unlike T lymphocytes, they do not express antigen-specific receptors or mediate antigen-specific immunity [6]. They form a heterogeneous group of cells that release type 2 cytokines, namely, IL-4, IL-5, IL-9, and IL-13[7]. Animal and clinical investigations confirmed that ILC2 modulates airway fibrosis, lung remodeling, and inflammatory responses in COPD [8, 9]. Moreover, although its quantity is rather limited in the lung, ILC2s critically link the innate and adaptive immune responses and maintain an intricate balance within the lung [10]. In a prior investigation, we demonstrated that ILC2 modulates the Th2 type immune response, which is critical for regulating the chronic COPD inflammatory response [11, 12]. Considering that ILC2s secrete type 2 and inflammatory cytokines, which strongly drive and exacerbate COPD, ILC2s targeting reagents may be highly beneficial in COPD therapy. Notably, it is essential to inhibit ILC2 proliferation, as well as the synthesis of the aforementioned cytokines. More importantly, a high-fiber diet or supplementation was shown to substantially attenuate emphysema and protect against COPD in both human and animal models, the mechanisms of which remain unclarified [13, 14, 15].

Commensal microbiota strictly modulates the immune system. Emerging evidences suggest the strong significance of microbiota-based short chain fatty acids (SCFAs) in regulating ILC functions [16]. SCFAs primarily originate from dietary fiber utilizing microbial fermentation, including acetate (C2), propionate (C3), and butyrate (C4) [17]. Butyrate suppresses ILC2 activity and ameliorates airway inflammation in murine models [10]. Additionally, butyrate diminishes GATA3 expression in ILC2 cells, thereby inhibiting lung ILC2 cellular activity, and reducing lung airway hyperresponse [18]. Moreover, it diminishes the metabolic status, oxidative phosphorylation level as well as the glycolytic pathway.

SCFAs also activates the G-protein-coupled receptors (GPRCs) signaling pathway and suppresses histone deacetylase (HDAC) activity. Emerging evidences revealed that both FFAR2 activation and HDAC suppression contribute to the SCFA-mediated regulation of Treg production [19, 20]. Nonetheless, Park et al. [21] reported that acetate, propionate, and butyrate enhance Th17 and Th1 effector cell synthesis *in vitro*, which in turn induce suppression of HDACs activity. C4 promotes macrophage anti-inflammatory activities in a GPR109-reliant fashion [22]. In a prior investigation, we revealed that butyrate diminishes iILC2 cellular content in the intestinal tissues of COPD mice, which, in turn, alleviates pulmonary immune inflammatory response [23]. Unfortunately, till date, the dietary fiber significance of butyrate action on ILCs, particularly ILC2s in context of COPD, remains unelucidated.

Herein, we demonstrated that butyrate, extracted from microbial high fiber diet fermentation, strongly inhibited ILC2-mediated type 2 cytokine formation *in vitro*. Moreover, it also relieved ILC2-based inflammation *in vivo*. Based on transcriptome analysis, the histone acetylation status in the NFIL3 promoter region was greatly enhanced following butyrate treatment. This ultimately suppressed ILC2 cell-based IL-5 and IL-13 release. Collectively, we demonstrated an intricate mechanism of a critical and well-established bacterial metabolite in gut function. Moreover, we also established its distal regulation of cells, namely, the lung tissue. This unique association also regulates immune reactivity via its modulation of innate lymphoid cells.

## 2. Results

### 2.1. High-Fiber Diet Modulates COPD Microbiota and Short-Chain Fatty Acid Content to Inhibit ILC2 Cell Function

**Figure 1:**
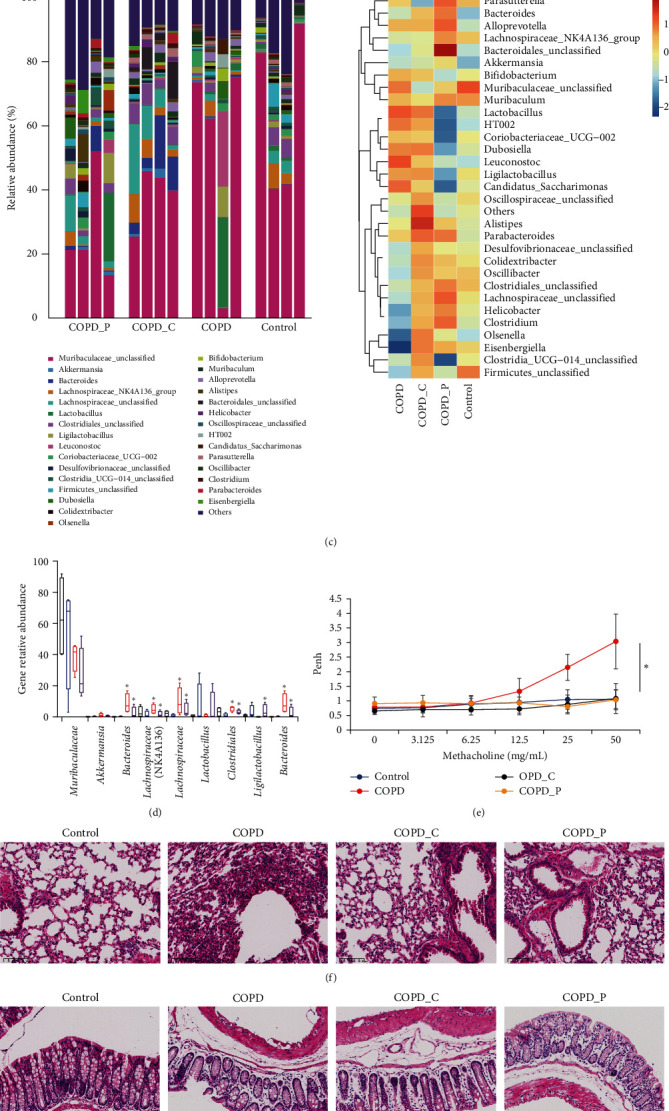
High-fiber diet suppresses COPD. We utilized cigarette smoke exposure to establish a COPD mouse model. COPD mice were fed with normal 4.5% cellulose chow (control diet) or 30% cellulose (Hi-Cellulose) or 30% pectin (Hi-Pectin) to serve as the fiber source. (a) An illustration of our study design. (b) Quantifications of colon propionate, butyrate, and valerianate amounts using GC-MS. nd indicates no detection. (c) Fecal pellet genus abundance using OTU count via 16S V4 profiling in individual mice, presented as as columns. (d) Mean most abundant bacterial genera contents. (e) The Penh value of mice given increasing methacholine doses. (f) H&E-stained lung tissue. (g) H&E-stained gut tissue. (h) Flow cytometric analysis of ILC2s in the whole lung. (i) CD45^+^Lin^−^CD127^+^GATA3^+^ILC2 cell. (j) Flow cytometric analysis of IL-13^+^ILC2s and IL-5^+^ILC2s in the whole lung. (k) Total IL-13^+^ILC2 in the Lung per mouse. (l) IL-5^+^ILC2 in the lung per mouse. (m) GATA3 transcript levels, as evidenced by qRT-PCR. Data presented as means ± SDs. (*n* = 15)  ^*∗*^*P*  < 0.05,  ^*∗∗*^*P*  < 0.01,  ^*∗∗∗*^*P* < 0.01, and  ^*∗∗∗∗*^*P*  < 0.0001 (relative to control mice).

High-fiber diet-derived short-chain fatty acids strongly reduce intestinal ILC2 cell recruitment to the lung, thereby alleviating lung-based allergic airway inflammation [18]. Nevertheless, the significance of dietary fiber in COPD remains unknown. Thus, herein, we examined whether dietary fiber regulates lung inflammation in COPD. To do this, we provided mice with normal 4%–5% cellulose chow (control diet) or 30% cellulose (Hi-Cellulose) or 30% Pectin (Hi-Pectin) diets of dietary formula, prior to establishment of a COPD mouse model (Figure 1(a)). Pectin fermentation is superior to cellulose, and it results in augmented SCFA synthesis *in vivo*. In general, the colon contains millimolar amounts of valerianate, propionate, and butyrate. We demonstrated that 2 months of pectin diet dramatically augmented all SCFA contents, including propionate and butyrate (Figure 1(b)).

Butyrate is preferentially produced by Firmicutes in mice, and it contains Clostridia IV and XIVa. Herein, we assessed alterations in microbiota of diet-modified mice via sequencing of 16S ribosomal RNA of murine fecal pellets. Based on the Chao1 index, the COPD mice intestinal species diversity was substantially diminished, whereas the quantity of intestinal microbiota species in the Hi-Cellulose and Hi-Pectin fed COPD mice was dramatically increased. In terms of phylogenetics, Hi-Pectin diet strongly upregulated bacteroides, as well as several distinct Firmicutes, such as genera Clostridiales and Lachnospiraceae, compared to the control or Hi-Cellulose diet, as evidenced by operational taxonomic unit (OTU) count (Figure 1(c)). As expected, we observed marked alterations in the contents of most abundant bacteria between controls and Hi-Pectin fed mice (Figure 1(d)), as evidenced by OUT. Together, these data suggest that a Hi-Pectin diet facilitates the proliferation of a specific microbiota linked to augmented SCFA abundance.

The COPD Penh values of control mice were substantially enhanced following methacholine challenge, indicating enhanced airway hyperresponsiveness and pulmonary airflow resistance. However, after 2 months of Hi-Pectin and Hi-Cellulose diet, the mice exhibited markedly reduced resistance and enhanced dynamic compliance, relative to COPD control diet mice (Figure 1(e)). Moreover, based on our HE staining, Hi-Pectin and Hi-Cellulose diet strongly improved pulmonary pathological alterations in COPD mice, as was evidenced by intact bronchial mucosal epithelium, and regularly arranged smooth muscle layer and submucosa (Figure 1(f)). The pathological results indicated that compared with COPD group, the degree of intestinal mucosal epithelial loss, erosion, lamina propria gland atrophy, and interstitial edema was reduced in Hi-Pectin and Hi-Cellulose diet groups, and the number of interstitial inflammatory cell infiltration was decreased (Figure 1(g)).Therefore, a hi fiber diet moderately but significantly alleviated COPD manifestations.

Type 2 innate lymphoid cell (ILC2) critically modulates COPD airway fibrosis, lung remodeling [8], and inflammation [24]. Cigarette smoke (CS) exposure strongly activates ILC2, thereby enhancing its contents in the lungs in the form of CD45^+^Lin^−^ (CD3, CD19, CD123, CD11b, CD11c, CD8, FceRI, CD14, CD4, and CD56) GATA3^+^CD127^+^. A pectin-rich diet dramatically reduced lung ILC2 content, relative to COPD mice (Figures 1(h) and 1(i)). To further assess ILC2 activity, we exposed total lung cells to brefeldin A for 4 hr to block cytokine release, and then we evaluated the intracellular cytokine formation on ILC2 using flow cytometry. Our findings revealed that although most activated ILC2 in COPD mice produced IL-5 and IL-13, the total quantity of cytokine-producing cells, particularly IL-13, was significantly downregulated in Hi-Pectin fed mice (Figures 1(j), 1(k), and 1(l)). Next, we explored GATA3 mRNA expression in the lung ILC2 of COPD versus Hi-Pectin diet mice and demonstrated marked reduction of GATA3 expression with a Hi-Pectin diet (Figure 1(m)). Collectively, these results suggest that diet-induced upregulation of SCFA contents *in vivo* can potentially regulate ILC2 activity to simultaneously suppress type II response and GATA3 expression.

#### 2.1.1. SCFAs Reduce Inflammatory Cell Characteristics in Gut ILC2 Cells

**Figure 2:**
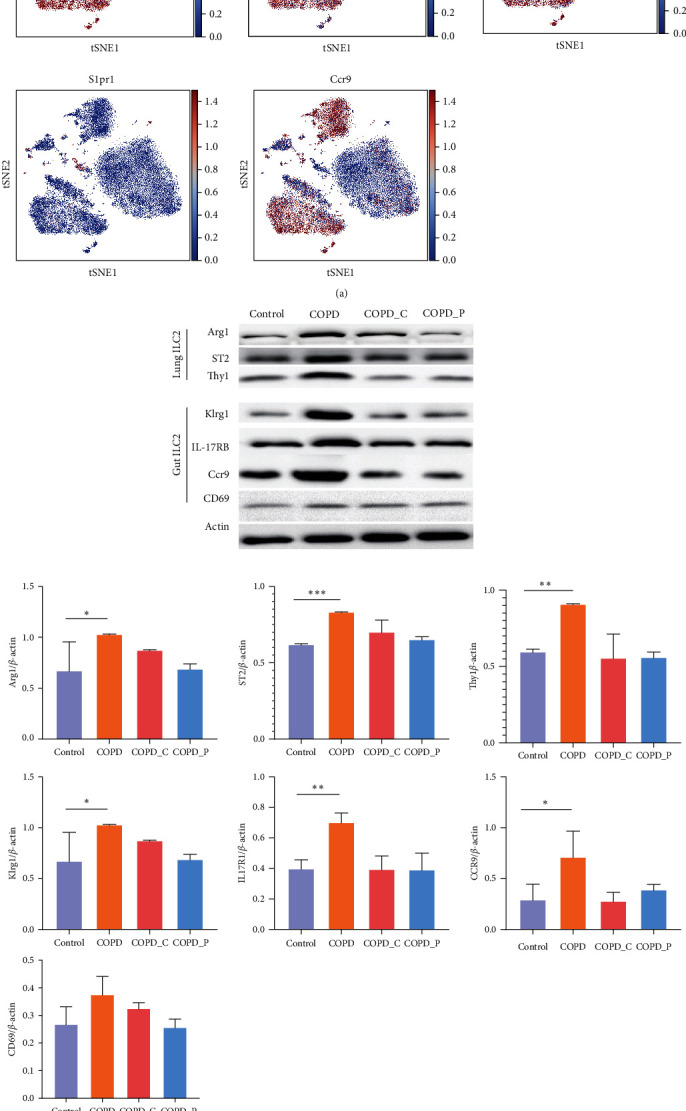
SCFAs alleviate inflammatory cell activities in gut ILC2 cells. (a) t-SNE feature plots revealing profiles of eight biomarkers. (b) Arg1, ST2, Thy1, KLRG1, IL-17RB, CCR9, and CD69 protein contents, as evidenced by Western blot analysis. (c) S1P, S1P2, S1P3, S1P4, and S1P5 transcript expressions, as evidenced by qRT-PCR. (d) Lung ILC2s mRNA expression in murine COPD model treated with butyrate.  ^*∗*^*P* < 0.05,  ^*∗∗*^*P* < 0.01, and  ^*∗∗∗*^*P* < 0.01 (relative to control mice).

We downloaded the murine ILC2 scRNA-seq information from the Gene Expression Omnibus (GEO) under accession code GSE117568. Using mining scRNA-seq data, we demonstrated that lung ILC2s contained copious amounts of ST2, Thy1, Arg1, with scarce expression of IL17RB, and KLRG1. In contrast, the gut ILC2s expressed large quantities of IL17RB, KLRG1, and Ccr9, with scarce expression of ST2, Thy1, and Arg1 (Figure 2(a)). Lung Thy1^hi^ nILC2s were endogenously derived; thus, they did not migrate from distal tissue/rec-number [25]. Several reports suggest that, under pathological conditions, intestinal KLRG1^hi^ iILC2s cells migrate from the distal intestine to the lung using the S1P network. In the lung, they release IL-5 and IL-13 and activate eosinophilic lung accumulation, calcination, mucus aggregation, epithelial interstitial reaction, and finally pulmonary airway hyperresponse and airway remodeling [8, 25, 26]. Moreover, Ccr9 expression is ubiquitous in gut ILC2 cells, whereby it behaves like a chemokine promoting cell migration to intestinal lamina propria [27].

To investigate the effects of a high-fiber diet on ILC2 cells in lung and gut ILC2 cells, we conducted WB. Our results revealed that ST2, Thy1, and Arg1 were strongly upregulated in lung ILC2 cells, whereas IL-17RB and Klrg1 were elevated in the intestinal tissues of COPD mice. A high-fiber diet, on the other hand, strongly diminished IL-17RB and Klrg1 contents in gut tissues, with simultaneous reduction in Ccr9 expression. In addition, the expression of CD69 was increased again in COPD group, but there was no statistical difference compared with other groups. Based on these results, high-fiber diet expression effectively decreased lung and gut ILC2 cell-specific gene expression (Figure 2(b)). Subsequently, we utilized qRT-PCR to explore alterations in gut ILC2 S1PR expression. We revealed that the gut ILC2 cells of COPD mice exhibited markedly elevated S1PR expression; however, a Hi-Cellulose diet and high-fiber diet diminished S1PRs levels (Figure 2(c)).

To further elucidate the significance of butyrate in lung and gut ILC2 cell inflammation, COPD mice were continuously administered 150 mM butyrate for 2 weeks, prior to transcriptome analysis. Based on our observation, ILC2 exhibited the largest rises in IL-5 and IL-13 transcription levels in COPD group. With butyrate treatment, there was over 50% decline in IL-5 and IL-13 levels, as is evidenced by heatmap of log^2^ absolute expression counts (Figure 2(d)). Butyrate also diminished IL-1rl1 (ST2), IL-2ra (CD25), IL-17, and icos surface markers, relative to control and COPD mice. Furthermore, our transcriptome analysis revealed that butyrate enhanced levels of negative regulators tnfaip3 (A20) and Ctla4. The transcription factors GATA3 and IRF4 critically regulate ILC2-mediated cytokine production [18, 28]. COPD mice ILC2 exhibited marked enhancements in GATA3 transcription levels. However, butyrate treatment diminished GATA3. Moreover, irf4 expression among COPD and butyrate-treated mice was diminished, relative to controls. This indicated that butyrate strongly reduced GATA3 in ILC2 (Figure 2(d)). Additionally, we revealed that butyrate strongly diminished Ccr9 transcripts. Together, these data indicated high-fiber diet treatment inhibits S1PR expression in ILC2s; their ability to exit not only the gut but also the lungs can be decreased.

#### 2.1.2. Butyrate Suppresses ILC2-Based Cytokine Formation In Vitro and In Vivo

**Figure 3:**
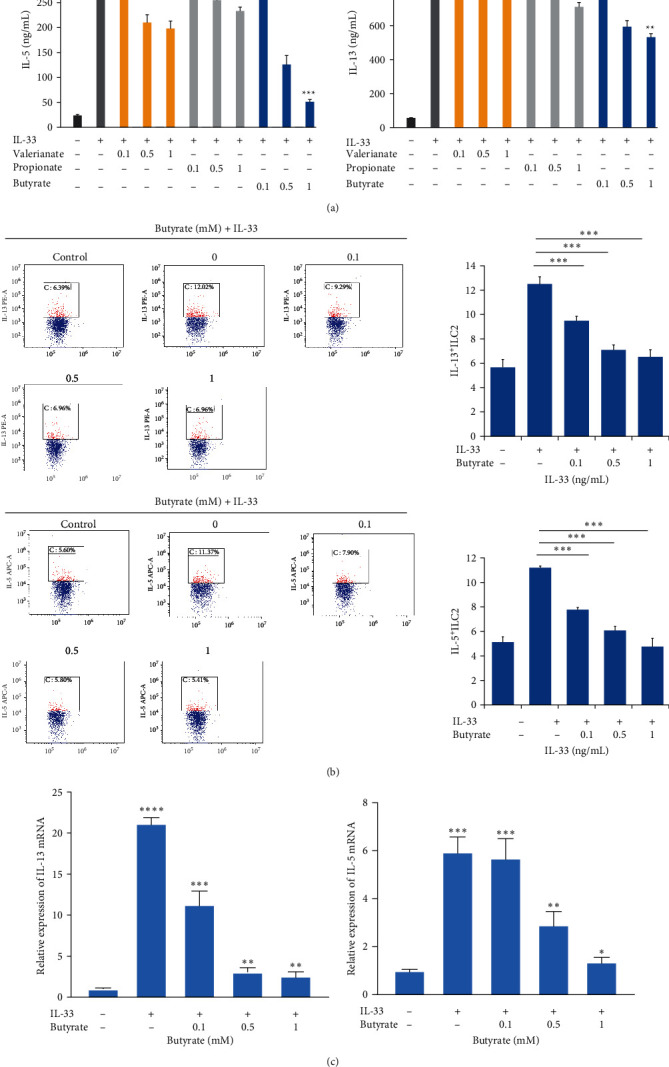
Butyrate, not acetate or propionate, suppresses cytokine production in ILC2s. (a) IL-13 and IL-5 contents in supernatant of SCFA-activated ILC2, as evidenced by ELISA. (b) An illustration of the intracellular profiles of IL-13 and IL-5 in ILC2s following 48 hr of butyrate exposure. (c) IL-13 and IL-5 transcript levels following 6 hr of intervention.  ^*∗*^*P* < 0.05,  ^*∗∗*^*P* < 0.01,  ^*∗∗∗*^*P* < 0.01, and  ^*∗∗∗∗*^*P* < 0.0001 (relative to control group).

To explore the SCFA-mediated regulation of ILC2 action, we evaluated the outcomes of valerianate, propionate, and butyrate on type 2 cytokine synthesis by IL-33-induced ILC2s. First, we sorted lung ILC2 cells using magnetic beads(STEMCELL, Vancouver, BC, Canada), and then we exposed the sorted ILC2s to SCFAs and IL-33 to show that butyrate, but not valerianate or propionate, strongly diminished IL-13 and IL-5 contents in the supernatant of cultured ILC2s in a dose-reliant fashion (Figure 3(a)).

Next, we examined whether this observation was due to impaired cytokine formation. To do this, we conducted intracellular IL-13 and IL-5 stainings in butyrate- and IL-33-treated lung ILC2 for 48 hr. We revealed that butyrate drastically diminished IL-13+ILC2s and IL-5+ILC2s contents, along with marked reductions in expressions of IL-13 and IL-5 mRNA (Figures 3(b) and 3(c)).

**Figure 4:**
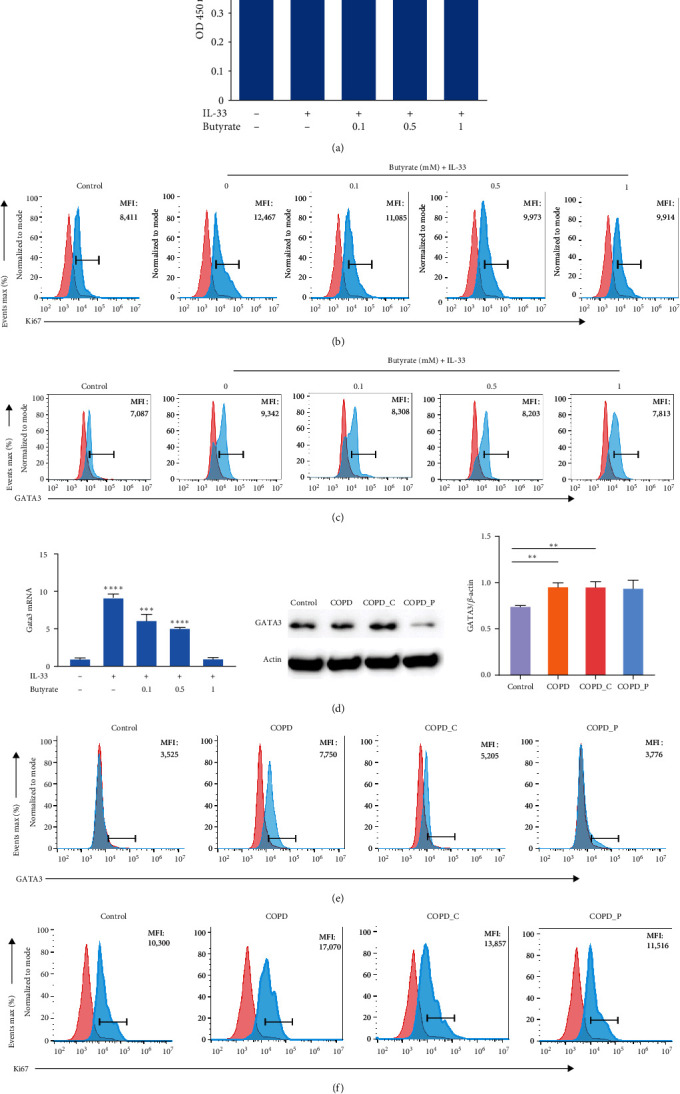
Butyrate suppresses ILC2 proliferation and GATA3 expression but not viability. (a) Cell survival, as evidenced by a Cell Counting Kit-8 assay. Optical density at 450 nm (*n* = 5). (b) ILC2 proliferation, as evidenced by Ki-67 following 48 hr of intervention (*n* = 5). (c) GATA3+ILC2s percentage following 48 hr of intervention. (d) Gata3 transcript levels following 6 hr of intervention (*n* = 5). (e) GATA3+ILC2s percentages in control, COPD, COPD_C, and COPD_P mice, respectively. (f) Ki-67+ILC2s percentages in control, COPD, COPD_C, and COPD_P mice, respectively.  ^*∗∗*^*P* < 0.01,  ^*∗∗∗*^*P* < 0.01, and  ^*∗∗∗∗*^*P* < 0.0001.

Emerging evidences revealed that butyrate inhibits cellular proliferation and enhances apoptosis in multiple immune and nonimmune cell types [10]. To assess whether butyrate similarly regulates ILC2s in COPD, we sorted lung ILC2s and treated then in medium containing IL-33 with or without butyrate. Using CCK-8 analysis, we demonstrated that butyrate did not stimulate marked apoptosis at concentrations of 1 mM or lower (Figure 4(a)).

We then assessed the proliferation rate of ILC2s using Ki-67 staining. As expected, IL-33 treatment increased the proliferation capacity of ILC2s when compared with unstimulated control cells, as indicated by the increase in the percentage of Ki-67 + cells. Butyrate treatment markedly decreased the percentage of Ki-67+ILC2s, causing near-complete inhibition at a dose of 0.5 mmol/L (Figure 4(b)).

Thereafter, we evaluated the influence of butyrate exposure on GATA3 expression in IL-33-activated and butyrate-treated ILC2s *in vitro*. Butyrate significantly diminished GATA3 levels, as was evidenced by reduced mean fluorescence intensity after 48 hr exposure in a dose-reliant fashion (Figure 4(c)). A 0.5 mM minimum dose was necessary for inhibition, and this corresponded to the dose required to suppress cytokine formation at the transcript and protein levels. Similarly, the GATA3 expression was also reduced with butyrate treatment (Figure 4(d)). Additionally, using qRT-PCR, we revealed strong downregulation of IL-13 and IL-5 mRNA expressions following butyrate exposure, which indicated negative regulation at the level of gene transcription (Figure 3(c)).

We next validated our results using a COPD mouse model. In all, we evaluated the lung ILC2 cells of control, as well as COPD with control diet, Hi-Cellulose, and pectin (Hi-Pectin) mice, respectively. As expected, we revealed a strong reduction of GATA3 expression in the butyrate-treated Hi-Cellulose and Hi-Pectin fed mice (Figure 4(e)). In the control mice, most lung ILC2s showed negative Ki-67 staining. In contrast, COPD mice exhibited enhanced Ki-67+ population (Figure 4(f)). Notably, COPD mice with Hi-Pectin showed drastic reduction in cellular population, as well as overall Ki-67+ ILC2s percentage.

#### 2.1.3. Butyrate Ameliorates GPCRs But Does Not Affect ILC2 Function

**Figure 5:**
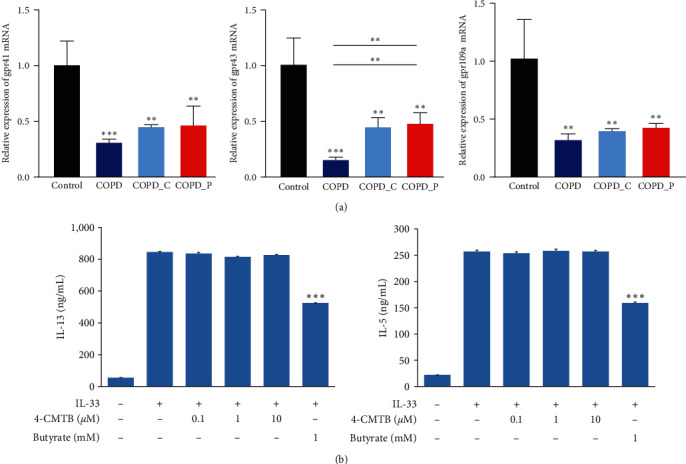
GPR43 agonist does not impair ILC2 function. (a) gpr41, gpr43, and gpr109a transcript levels, as evidenced qRT-PCR. (b) Sorted lung ILC2s exposed to 0.1, 1, and 10 *μ*M TSA, 1 mM butyrate, or both, along with IL-33 (10 ng/mL) for 48 hr. IL-13 and IL-5 contents in culture supernatants of ILC2s, as evidenced by ELISA.  ^*∗*^*P* < 0.05,  ^*∗∗*^*P* < 0.01, and  ^*∗∗∗*^*P* < 0.01.

SCFAs are known to utilize GPCRs GPR41, GPR43, and GPR109a for signaling [29]. We evaluated the gpr41, gpr43, and gpr109a transcript expressions in ILC2 cells using qRT-PCR. We revealed strong downregulations of gpr41, gpr43, and gpr109a transcript contents in ILC2 cells in COPD mice; however, these mRNAs were markedly enhanced in the Hi-Cellulose and Hi-Pectin fed mice. Among them, gpr43 mRNA was particularly elevated in the Hi-Pectin fed mice (Figure 5(a)). To explore the significance of gpr43 activation on ILC2 activity, we exposed IL-33-activated ILC2s to 4-chloro-alpha- (1-methylethyl)-N-2-thiazoylylbenzeneacetanilide (4-CMTB) (Tocris Bioscience, Bristol, UK), which served as a GPR43 agonist at 0.1, 1, and 10 *μ*mol/L (*μ*M) concentrations, and assessed the supernatant cytokine levels. Unlike butyrate, 4-CMTB exposure failed to reduce IL-33-induced IL-13 and IL-5 levels. Moreover, 4-CMTB treatment had no effect on the butyrate-mediated inhibition of IL-13 and IL-5 synthesis by ILC2s (Figure 5(b)). Taken together, these data revealed that GPR43 activation does not influence ILC2 activity; therefore, the butyrate-mediated inhibition is potentially independent of GPR.

#### 2.1.4. Butyrate-Mediated Inhibition Employs HDAC Inhibition

**Figure 6:**
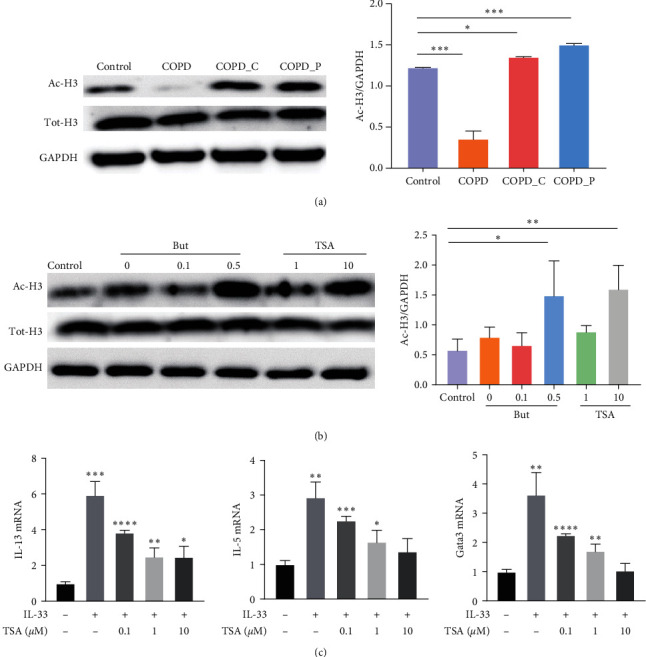
Butyrate inhibits ILC2 activity via HDAC inhibitory action. (a) Typical western blot image of ac-H3 level in ILC2 cells in lung tissue of control, COPD, COPD_C, and COPD_P mice, respectively. (b) Sorted lung ILC2s exposed to 0, 0.5, and 1 mM butyrate and 0.1, 1, and 10 *μ*M TSA, along with IL-33 (10 ng/mL) for 48 hr, The ac-H3 status, as evidenced by western blot. (c) Sorted lung ILC2s exposed to 0.1, 1, and 10 *μ*M TSA, along with or without IL-33 (10 ng/mL) for 48 hr, the relative expression level of IL-13 and IL-5 mRNA, as evidenced by qRT-PCR.  ^*∗*^*P* < 0.05,  ^*∗∗*^*P* < 0.01,  ^*∗∗∗*^*P* < 0.01, and  ^*∗∗∗∗*^*P* < 0.0001.

Butyrate serves as an HDAC inhibitor and promotes histone and non-histone protein acetylation in numerous cell types. To assess whether butyrate behaves as an HDAC inhibitor in ILC2s, sorted lung ILC2s from the control, as well as COPD with control diet, Hi-Cellulose, and Hi-Pectin mice and associated H3 acetylation, were closely evaluated. We revealed that in COPD mice, the H3 acetylation status was diminished. In contrast, both Hi-Cellulose and Hi-Pectin fed mice exhibited enhanced H3 acetylation relative to the COPD mice (Figure 6(a)).

We next explored the relevance of HDAC inhibition in ILC2 function. To do this, we exposed IL-33-activated ILC2s to rising TSA concentrations. Similar to our observations with butyrate, the H3 acetylation was substantially increased with TSA treatment in a dose-reliant fashion (Figure 6(b)). Moreover, the IL-13 and IL-5 transcript and protein levels were suppressed by TSA treatment in a dose-reliant fashion (Figure 6(c)). Additionally, the GATA3 expression was also diminished (Figure 6(c)).

#### 2.1.5. Butyrate Enhances NFIL3 Expression While Suppressing IL-5 and IL-13 Contents

GATA3 is a major contributor to the ILC2-mediated production of IL-5 and IL-13 cytokines. Prior investigations reported that NFIL3 inversely modulates IL-13 and IL-5 expressions [30].

**Figure 7:**
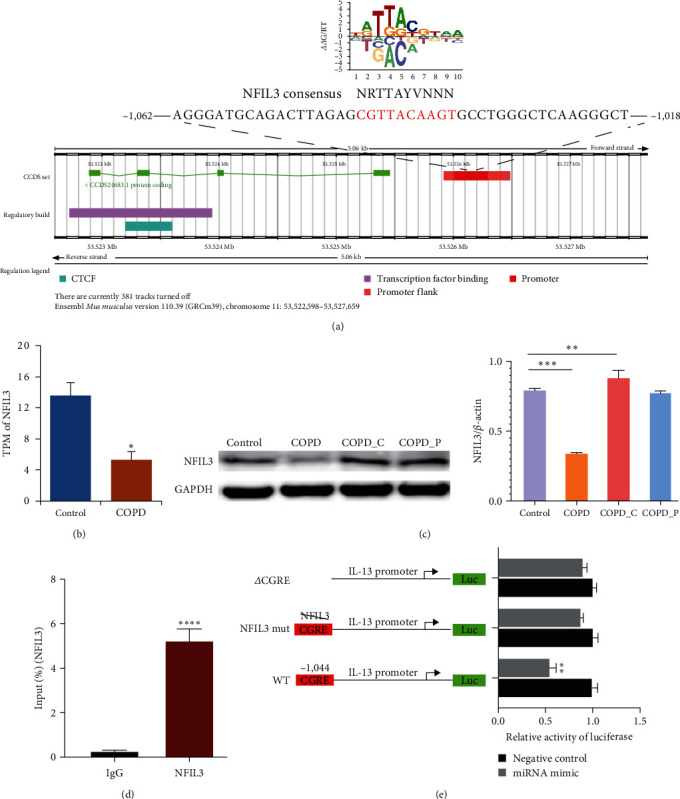
NFIL3 interacts with CGRE to suppress IL-13 gene expression. (a) An illustration of the NFIL3-interacting and consensus NFIL3-interacting sequences. (b) TPM of nfil3 in ILC2 cells in the lung tissue of control and COPD mice. (c) NFIL3 content in ILC2 cells within the lung tissues of control, COPD, COPD_C, and COPD_P mice, respectively. (d) Sorted lung ILC2s were crosslinked, prior to immunoprecipitation of soluble chromatin complexes using anti-ac-H3 antibody or control IgG. PCR-amplified region including CGRE in the coprecipitated DNA. *β*-Actin served as the negative control. (e) Negative modulation of the IL-13 gene transcription via NFIL3. The reporter constructs of WT, NFIL3-interacting mutant, and CGRE-deletion mutant, as evidenced by luciferase assay.  ^*∗*^*P* < 0.05,  ^*∗∗*^*P* < 0.01,  ^*∗∗∗*^*P* < 0.01, and  ^*∗∗∗∗*^*P* < 0.0001.

Based on the JASPAR database of NFIL3 binding motifs, NFIL3 associates with a region upstream of the first exon of IL-13 gene 1.0–1.1 (evolutionarily conserved sequence 1.0–1.1 kb upstream of the first exon) (Figure 7(a)). Herein, we revealed that the NFIL3 expression was significantly diminished in COPD mice (Figure 7(b)). In addition, we detected that the expression of NFIL3 protein was decreased in the COPD group and increased in the high-diet group (Figure 7(c)). The H3 acetylation status-based Chip-PCR results revealed an augmented acetylation status in the NFIL3 promoter of ILC2 cells in the high-fiber diet fed mice only (Chip-PCR data with IgG control) (Figure 7(d)).

To better explore the link between the NFIL3 protein and IL-13 transcriptional activity, we incorporated ILC2 cells with pGL3-il13-CGRE-WT, pGL3-il13-CGRE-MUT, and pGL3-il13*Δ*CGRE for 48 hr, prior to extraction of cellular lysates for luciferase assay. Relative to WT mice, mutation within the NFIL3-interacting site strongly upregulated IL-13 transcriptional activity. Collectively, these data indicated that the NFIL3-interacting site in the CGRE region sometimes functions to regulate IL-13 transcription.

## 3. Discussion

Emerging evidences revealed that gut microbiome is impaired in COPD patients, who also experience reduction in intestinal microbial diversity as well as dysfunctional immune system, which contribute to a chronic inflammation status [31, 32, 33]. Multiple reports suggested that gut microbial metabolites are critical for regulation of health maintenance, pathogen colonization, and immune response [34, 35, 36]. Based on some reports, ILC2 strongly modulates lung homeostasis, lung structural repair/remodeling post injury, and inflammation activation, along with other intricate functions during immune response [37]. Prior investigation by us and other scientists demonstrated that gut microbial metabolites diminish ILC2 cellular function [23, 38]. Nonetheless, the significance of microbial metabolites ILC2-induced COPD inflammation regulation remains unknown. The findings from this study indicated that the intestinal microbiota metabolite butyrate strongly controlled ILC2 cell activity to alleviate COPD-based lung inflammation.

Dietary fiber fermentation-derived SCFAs, especially acetate, propionate, and butyrate [39], are known to improve innate immune-induced CS-COPD [40]. COPD patients are typically deficient in fiber intake [41] and have reduced lung function and advanced disease progression. Based on earlier animal experimentations, SCFA synthesis is upregulated with fecal microbiota transplantation (FMT) and high-fiber diet (HFD) and provides protection against COPD disease in mice. Dietary fiber is intricately linked to enhanced lung function and diminished COPD prevalence [41]. Similarly, we revealed that high-fiber diet with butyrate infusion in drinking water inhibited the cardinal characteristics of smoke-induced inflammation, such as lung function, and TH2 cytokine release, via suppression of lung ILC2 expansion. Moreover, high-fiber diet also introduced multiple alterations in gut microbiome, which accelerated the pathogenesis and progression of COPD. For instance, COPD patients express scarce amounts of Bacteroidetes, relative to healthy controls. Moreover, the SCFAs level in COPD III–IV patient stools was strongly diminished, relative to other cohorts [42]. Hopkins et al. [43] reported that butyrate decreases CS-COPD lung damage via inhibition of the mevalonate axis in the gut, lungs, and liver. Mao et al. [44] revealed that the Bufei Jianpi formula (BJF) augmented Firmicutes population, as well as the Firmicutes- to-Bacteroides ratio, thereby raising SCFA contents, improving pulmonary function, and alleviating lung inflammation in COPD rats [44]. Given the aforementioned evidences, our study provided further proof that the gut metabolite production strongly influenced airway inflammation in COPD.

SCFAs modulate immune cell proliferation, differentiation, and function [45]. Several investigations revealed that SCFAs augmented Foxp3 histone H3 acetylation to accelerate Treg cell generation [46, 47]. Additionally, SCFAs suppressed allergic inflammation by altering the fate of naive CD4+T cells from Th9 cells to FoxP3+ Tregs [48]. Moreover, the naïve IFN*γ*+T, ILC2, and ILC3 cell differentiation was also inhibited by SCFAs in other studies [10, 18, 49]. Although mounting evidence suggests that butyrate can suppress the proliferation and foster apoptosis of various immune and nonimmune cells [50] but study showed that butyrate (<1 mM) did not augment the percentage of apoptotic cells [10]. Herein, we revealed that SCFAs, particularly butyrate, diminished ILC2 quantities in the lung and gut of COPD mice. Furthermore, *ex vivo* IL-33 exposure expanded lung ILC2s in response to butyrate (0.5–1 mM), thus revealing a dose-reliant reduction of IL-13 and IL-5 gene expression and cytokine secretion.

Given that colon epithelial cells can absorb large amounts of SCFA, the total SCFA quantity in the intestinal lumen is typically about 100–150 mM, while the SCFA concentration in the bloodstream remains low, usually between the range of 0.1–1 mM [39]. Hence, we fed mice with 150 mM butyrate in drinking water, a concentration used in prior publications [10, 51], for 2 weeks during the acute exacerbation of COPD. We revealed that butyrate drastically decreased IL-5 and IL-13 mRNA in ILC2 cells. Additionally, the receptor molecules IL-1rl1 (ST2), IL-2ra (CD25), and IL-17 of ILC2 cells in COPD mice were substantially reduced following butyrate treatment, whereas the negative regulatory molecules tnfaip3 and Ctla4 were elevated. ILC2 requires the transcription factors GATA3, ST2, and IL-7r, whereas GATA3 is required for ILC2 to persist *in vivo* and produces IL-5 and IL-13, and butyrate diminishes GATA3 in ILC2. Based on our observations, butyrate treatment strongly decreased ccr9 transcripts in COPD mice. According to other studies, SCFAs are robust anti-inflammatory chemicals that reduce immune cell adhesion and chemotaxis while increasing anti-inflammatory cytokine production and cellular apoptosis. To suppress lung inflammation, it is therefore possible to prevent intestinal ILC2 cells from spreading to the lungs.

Butyrate signals through the G protein-coupled receptor (GPCR) class, namely, GPR41/FFAR3, GPR43/FFAR2, and GPR109A/HCAR2 [52]. SCFA-GPR networks are known to strongly modulate immune responses. GPR41 and GPR43 are abundant on immune cell lineages, whereas GPR109a is activated primarily by butyrate and niacin [53]. Herein, we examined the significances of SCFAs and their receptors in modulating lung ILC2s in COPD mouse. We revealed that although GPR43 was scarcely expressed in COPD mice, Hi-Pectin diet-induced microbial metabolites both promoted and activated GPR43. Moreover, based on our *in vitro* activation and blocking results, butyrate may not utilize GPCR receptor activity to suppress ILC2 cellular function.

SCFAs are also reported to control HDAC activity. Herein, we revealed that the acetylated H3 status was markedly diminished in the lung ILC2 cells of COPD mice. However, H3 acetylation was enhanced in Hi-Cellulose and Hi-Pectin fed mice. Furthermore, both butyrate and TSA, a pan-HDAC inhibitor, upregulates acetylated H3. TSA is known to block ILC2-mediated allergic inflammation in mice via reduction the lung ILC2s population [54]. Consistent with these evidences, in this study, we revealed that histamine H3 acetylation status was enhanced in ILC2 cells in TAS and butyrate-treated, whereas the mRNA levels of IL-13 and IL5 as well as nuclear transcription factor GATA3 were decreased. Meanwhile, IL-13 and IL-5 secretion also decreased. The evidences suggested that butyrate served as an HDAC inhibitor in ILC2s.

Histone acetylation opens up the chromatin structure, thereby facilitating cis-regulatory element access to basal transcription factors, chromatin remodelers, and RNA polymerase II [55]. However, based on our results, butyrate/TSA suppressed ILC2 cellular function; however, the level of total histone acetylation was markedly enhanced. Histone acetylation modification is known to recruit chromatin-repressive complexes, such as Mi-2/NURD, to mediate transcriptional inhibition in macrophages [56]. NFIL3 is as a crucial transcriptional modulator controlling immune cell development and differentiation. Studies revealed that NFIL3 negatively regulates hepatic gluconeogenesis [57], in myeloid cells. NFIL3 is IL-10 inducible and serves a primary function as a IL-12p40 transcriptional repressor that suppresses inflammation in colitis [58]. Hence, in this study, we examined whether the butyrate/TSA-mediated inhibition was mediated by NFIL3 via the transcriptional activation/inhibition of Th2-related genes. We demonstrated that NFIL3 was strongly suppressed in COPD mice and was enhanced in high-fiber diet mice. Moreover, using H3 acetylation Chip-PCR analysis, we revealed that the NFIL3 promoter acetylation was markedly upregulated in ILC2 cells in the high-fiber diet mice, which, in turn, promoted NFIL3 expression. NFIL3 serves as a transcriptional suppressor to inhibit IL-13 expression. Kashiwada et al. reported that NFIL3 strongly modulates chromatin remodeling of the TH2 cytokine locus, thereby downregulating Th2-related gene expressions [30].

## 4. Materials and Methods

### 4.1. Mice

In all, we obtained 60 SPF male C57BL/6J mice, 6 weeks of age, from the Experimental Animal Center of Xinjiang Medical University, and housed them at (22–25)°C, with 45–65% relative humidity, 12/12 hr light/dark period, and open access to drinking water. Following 1 week of conditional feeding, the animals were arbitrarily separated (using a random number table procedure) into two cohorts, namely, control group (CTL, room air inhalation, no CS, *n* = 15) and cigarette smoke group (CS, *n* = 45). Subsequently, CS mice were further separared into three cohorts, namely, normal chow, 4%–5% cellulose fiber content (w/w), and 30% cellulose and 30% pectin diet groups, with 15 mice per cohort. A COPD model was developed by placing mice inside an in-house designed glass smoker (120 × 80 × 60 cm^3^) for days 1–180 to introduce cigarette exposure (9 cigarettes/hour, 2 hr/time, 2 times/time, and 6 days/week). The employed cigarette type was the Snow Lotus brand produced by the Xinjiang Cigarette Factory in China (coke 12 mg, smoke nicotine 1.0 mg, and smoke CO 13 mg). We next monitored daily alterations in the murine hair color and behavior during model establishment and recorded murine weight and lung function post model establishment. Mice euthanization utilized carbon dioxide (RC-1001-15L, Shanghai Yuyan Instrument Co., Ltd., China), and serum, lung, and intestinal tissues were collected for analyses. This work received ethical approval from the Xinjiang Medical University.

### 4.2. Airway Responsiveness Evaluation

Following COPD establishment, we recorded airway methacholine (Sigma–Aldrich, St. Louis, MO, USA) responsiveness via a noninvasive pulmonary function instrument (Fine-Pointe NAM system; St. Paul, MN, USA), as reported previously [23].

### 4.3. 16 S rRNA Gene Sequencing

We assessed fecal 16S rRNA genes using Illumina MiSeq (CA, USA) to identify intestinal microbiota composition, as reported previously [23]. Data are uploaded as SRA.

### 4.4. SCFA Recording

SCFA content was recorded using gas chromatography–mass spectrometry (GC–MS), as reported previously [23]. The concentration of SCFA was calculated according to the standard curve.

### 4.5. Flow Cytometry

ILC2 cells in lung and intestinal tissues were sorted using the following method. Approximately, 30 mg of lung tissue was carefully sliced into 1–2 mm^3^ small pieces, prior to a 1-min homogenization in an ice bath. For intestinal tissue, longitudinally dissect, rinse with ice PBS (pH 7.4), and cut into small pieces after eliminating Peyer's patches. Epithelial cells were removed by incubating the tissue for 30 min at 37°C with constant stirring at 350 rpm in extraction buffer (5% fetal bovine serum (FBS), 1 mM DTT, and 10 mM EDTA in PBS).

Thereafter, 1 mg/mL preheated (37°C) fresh type IV collagenase (Sigma–Aldrich, St. Louis, MO, USA) was added to the pretreated intestinal and lung tissues, followed by DNA digestion using 40 *μ*g/mL DNAse I (Sigma–Aldrich, St. Louis, MO, USA) in RPMI-1640 culture solution in a 37°C water bath with gentle shaking every 3–5 min for 60 min total. Digestion was terminated via ice bath, and scattered cells were gently blown to collect the digestive fluid, which was then put through a 200 mesh filter, prior to a 5-min centrifugation at 1,000 rpm. The resulting pellet was PBS-rinsed, prior to the addition of 5-mL red cell lysate, and a 10-min incubation on ice. The suspension was centrifuged again to remove supernatant and then PBS-rinsed and blown, before centrifugation, 1–2 more PBS rinses, discarding of supernatant, and finally addition of 2 mL PBS containing 1% BSA at 4°C to achieve the murine lung single-cell suspension.

Lung single-cell suspensions underwent ILC2 staining as Lineage^−^CD45^+^CD127^+^GATA3^+^. To conduct transcription factor staining, we employed the FOXP3 fixation permeabilization kit, as directed in associated protocol, prior to GATA3-PE (eBioscience San Diego, CA) staining. To detect cytokines, cells underwent a 4-hr stimulation with PMA Ionomycin and brefeldin A (Cell Stimulation Cocktail, Biolegend) or brefeldin A alone, followed by cell fixation and intracellular cytokine staining for IL-5-BV421 and IL-13-APC (Biologend, San Diego, CA), as directed by associated protocols. The resulting data were assessed via the Kaluza software (Beckman Coulter, Inc).

### 4.6. ILC2 Cell Sorting and Intervention

A single-cell suspension of mouse lung was prepared using the same method as before. The suspension was adjusted to 1 × 10^8^/mL, and ILC2 cells were isolated using magnetic beads (from stem cells and then BC, Canada); the purity of the separation was >95%. Next, the ILC2 cells were inoculated into 96-well plates (500 cells/well) with 10% FBS, 500 ng/mL of IL-2 (PeproTech, Rocky Hill, NJ, USA), 500 ng/mL of IL−25 (R&D Systems, Minneapolis, MN, USA), 500 ng/mL of IL-33 (R&D Systems, Minneapolis, MN, USA), and TSLP (PeproTech, Rocky Hill, NJ, USA) in IMDM medium for 5 days. After that, 5 × 10^3^ ILC2 cells were inoculated onto a 96-well plate. The cells were rinsed twice with PBS to eliminate the effects of cytokines and then treated with PBS and SCFAs (Sigma–Aldrich butyrate, St. Louis, MO, USA), respectively.

### 4.7. Quantitative Real-Time PCR (qRT-PCR)

Trizol LS (Takara, Tokyo, Japan) was employed for total RNA isolation from lung ILC2s. Then, the cDNA was synthesized from the total RNA using the Transcriptor First-Strand cDNA Synthesis Kit (Takara, Tokyo, Japan). Thereafter, we evaluated the mRNA levels of *Il13*, *Il5*, *Gpr109a*, *Gpr41*, *Gpr43*, *gata3*, *S1p*, *S1p2*, *S1p3*, *S1p4*, S1*p5*, and *nfil3* using qRT-PCR via the Bio-Rad CFX96 detection system (Bio-Rad, Hercules, CA, USA). The employed primers are summarized in Table 1, and the PCR parameters were as follows: 95°C for 5 min, then 40 cycles of 95°C for 30 s, and 60°C for 30 s. Target gene expression was computed using the 2^−*ΔΔ*CT^ formula, and *β*-actin served as the endogenous control.

### 4.8. Western Blot

Using cold RIPA buffer (Thermo Fisher Scientific, Waltham, MA, USA), we lysed gut or lung ILC2 cells to prepare for total protein isolation. Totally, 25 *μ*g protein was separated by 12% SDS-PAGE, which was then transferred onto the polyvinylidene fluoride membrane. The membrane was blocked with 5% nonfat milk at room temperature for 2 hr and further incubated overnight with rabbit anti-arginase1 (anti-Arg1) (Sangon Biotech, Shanghai, China), Thy.1 monoclonal antibody (Proteintech Group, Inc, Wuhan, China), ST2 monoclonal antibody (Proteintech Group, Inc., Wuhan, China), rabbit antikiller cell lectin-like receptor G1 (KLRG1) (Abcam, Cambridge, MA, USA), rabbit anti-IL17RB antibody (bioss, Bejing, China), anti-CCR9 antibody (BosterBio, USA), rabbit anti-CD69 polyclonal antibody (absin, Shanghai, China), NFIL3 polyclonal antibody (Proteintech Group, Inc., Wuhan, China), rabbit anti-GATA3 polyclonal antibody (absin, Shanghai, China), histone-H3 polyclonal antibody (Proteintech Group, Inc., Wuhan, China), rabbit anti-acetyl-histone H3 monoclonal antibody (absin, Shanghai, China), or *β*-actin polyclonal antibody (Proteintech Group, Inc., Wuhan, China) at 4°C. Membranes were rinsed and then again incubated in corresponding secondary antibody (Abcam, Cambridge, MA, USA), prior to protein visualization via an enhanced chemiluminescence reaction kit (Cell Signaling Technology, Danvers, MA, USA). Protein quantification utilized Quantity One 1-D analysis software package (Bio-Rad, Hercules, CA, USA).

### 4.9. Chromatin Immunoprecipitation (ChIP)-PCR Analysis

ChIP was conducted under native conditions. In short, cells were cross-linked with formaldehyde, and then chromatin was fragmented by sonication. Following chromatin immunoprecipitation using specified antibodies or control IgG, co-IP DNA was purified, prior to quantification using aforementioned qRT-PCR. Relative gene expression computation utilized the Ct values and is presented as adjusted input (%) to the control IgG values. The employed NFIL3 primers are summarized in Table 1. The IP antibody was anti-acetyl Histone H3 (absin, Shanghai, China).

### 4.10. Dual-Luciferase Assay (DLA)

We developed luciferase reporter plasmids using pGL3-basic (Promega), with pRL-TK (Promega) as endogenous control. Quik Change Site Directed Mutagenesis Kit (Stratagene), and the following NFIL3-interacting mutant primers, 5′-TATTCCCCATAATTTAGGCTCCTCCTA-3′and 5′ACAGGAGGGATGCAGACTTAGAGCCGGGAGGTA-3′, were used to introduce mutations to the aforementioned reporter plasmid. NFIL3-overexpressing plasmid and its control were then coincorporated into cells to form wild-type (WT, pGL3-il13-CGRE-WT), mutant (MUT, pGL3-il13-CGRE-MUT), and CGRE deletion (*Δ*CGRE, pGL3-il13*Δ*CGRE) cells, respectively. Following a 48-hr incubation, cells were rinsed and lysed, and luciferase activities were assessed via the Dual Glo Luciferase Assay System (Promega), using directions from associated protocol.

### 4.11. ELISA

We recorded the IL-13 and IL-5 contents in the cell supernatant using corresponding ELISA kits (Lianke Biotechnology Co., Ltd., Hangzhou, Zhejiang, China), as per kit directions. Optical density (OD) was measured at 450 nm using a microplate reader.

### 4.12. Statistical Analysis

Data analyses utilized Graphpad Prism 8 (Graphpad Software Inc., San Diego, CA, USA) and are provided as mean ± SDs. One-way ANOVA, followed by LSD *t*-test, assessed multigroup differences. *P* < 0.05 was set as the significance threshold.

## 5. Conclusions

In summary, herein, we demonstrated that dietary-derived butyrate strongly inhibited ILC2 and subsequently alleviated lung inflammation in a COPD mouse model. Our evidences suggested that butyrate suppressed ILC2 proliferation and cytokine production potentially via suppression of GATA3 expression. Mechanistically, we confirmed that butyrate mediated its action via blockage of HDAC activity in a GPR-independent manner and via increase of the NFIL3 promoter acetylation status. Enhanced NFIL3 expression negatively influenced ILC2 cells to vastly reduce IL-13 release. This work presents the significance of butyrate as an immunomodulatory metabolite that controls COPD inflammation. Our findings suggest that diet-induced butyrate, as well as other microbial metabolites, can effectively prevent lung inflammation of COPD.

## Figures and Tables

**Table 1:** Quantitative RT-PCR using gene primers.

Gene	GenBank accession	Forward primer (5′→3′)	Reverse primer (5′→3′)	PCR product size (bp)	Tm (°C)
m-Il13	NM_008355.3	CTCTTGCTTGCCTTGGTGGTCTC	GGGAGTCTGGTCTTGTGTGATGTTG	140	60
m-Il5	NM_010558.1	AGAGAAGTGTGGCGAGGAGAGAC	CCATTGCCCACTCTGTACTCATCAC	96	60
m-Gpr109a	NM_030701.3	GGACAGACATGCCAAGATCAAGAGG	GTAGAGAAGCCAGAAGATGCGGATG	114	60
m-Gpr41	NM_001033316.2	CCACACTGCTCATCTTCTTCGTCTG	ACGGACTCTCACGGCTGACATAG	200	60
m-gpr43	NM_001168509.1	CTGTATGGAGTGATCGCTGCTCTG	CTGCTCTTGGGTGAAGTTCTCGTAG	112	60
m-Gata3	NM_001355110.2	TCTGGAGGAGGAACGCTAATGGG	CGGGTCTGGATGCCTTCTTTCTTC	112	60
m-S1p	NM_007901.5	CTGACCTTCCGCAAGAACATCTCC	CCCAGCAGGCAATGAAGACACTC	106	60
m-S1p2	NM_010333.4	CGCCATCGTGGTGGAGAATCTTC	GCCAGGTTGCCAAGGAACAGG	93	56
m-S1p3	NM_010101.4	GTTGGTGTGCGGCTGTCTAGTC	CTTCGGAGAGTGGCTGCTGTTG	118	60
m-S1p4	NM_010102.2	TACTCCTTCCGCAGCCGTGAG	TCGCAGACCTAGCCAGAGACAG	78	56
m-S1p5	NM_053190.2	TTGTGCTGGAGAACTTGGCTGTG	GTAGGATGTTGGTGGCGTAGGC	135	60
m-nfil3	NM_017373.3	CATTCCTCCCTCCCTCCTTTCTC	TGTGATGCCAGTGTTCCGATTTG	79	56
*β*-Actin	—	CATGTACGTTGCTATCCAGC	CATGTACGTTGCTATCCAGC	138	60

## Data Availability

The 16S rRNA gene sequencing data were deposited in NCBI with accession number of PRJNA1043400 (https://www.ncbi.nlm.nih.gov/sra/PRJNA1043400).

## Supplementary Materials

Supplementary Materials Figure S1 shows the flow cytometry gate strategy for ILC2.
