# Supplementary figures and images for: Dietary Fiber-Derived Microbial Butyrate Suppresses ILC2-Dependent Airway Inflammation in COPD
Source: Mediators Inflamm. 2024 Jul 9;2024:6263447. doi: 10.1155/2024/6263447 (PMC11251798; doi:10.1155/2024/6263447)

Supplementary Fig1

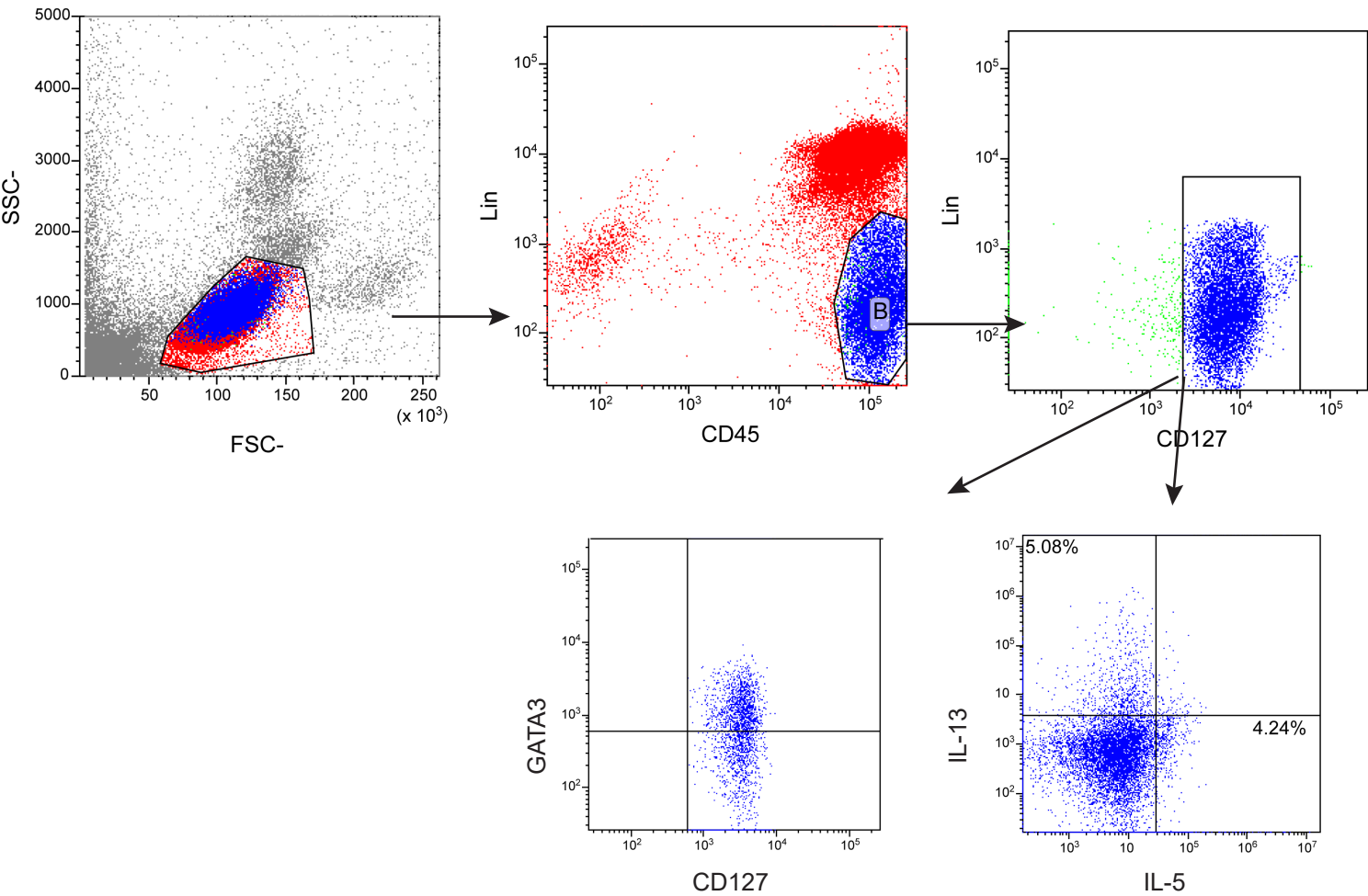

Supplement: Supplementary Materials — Figure S1 shows the flow cytometry gate strategy for ILC2. [file 6263447.f1.pdf]
